# EGFRVIII and EGFR targeted chimeric antigen receptor T cell therapy in glioblastoma

**DOI:** 10.3389/fonc.2024.1434495

**Published:** 2024-09-19

**Authors:** Robert C. Sterner, Rosalie M. Sterner

**Affiliations:** ^1^ Department of Neurosurgery, Inova Fairfax Medical Campus, Fairfax, VA, United States; ^2^ Department of Laboratory Medicine and Pathology, Mayo Clinic, Rochester, MN, United States

**Keywords:** epidermal growth factor receptor, EGFR, epidermal growth factor receptor variant III, EGFRvIII, chimeric antigen receptor T cell, CAR T cell, glioblastoma, solid tumor

## Abstract

Glioblastoma is the most common primary brain tumor. Although there have been significant advances in surgical techniques, chemo and immunotherapies, and radiation therapy, outcomes continue to be devastating for these patients with minimal improvements in survival. Chimeric antigen receptor T cell therapy is a revolutionary approach that is a new pillar in the treatment of cancer. CAR T cell therapy has produced remarkable results in hematological malignancies; however, multiple limitations currently prevent it from being a first-line therapy, especially for solid tumors. Epidermal growth factor receptor is classically amplified in glioblastoma, and a variant, EGFR variant III, is expressed on glioblastoma, making it an exciting potential target for CAR T cell therapy. Although preclinical has exciting potential, clinical data has been heterogeneous. In this review, we assess the state of field of EGFR-targeted CAR T cells.

## 1 Introduction

Glioblastoma multiforme (GBM) is the most common primary brain tumor that affects over 17,000 people annually (1, 2). Despite extensive efforts to develop numerous novel therapies, GBM continues to produce poor outcomes, with an average 5-year survival of 6.9% and a mean survival of 15 months (3, 4). Even with standard-of-care therapies including surgical resection, temozolomide, and radiotherapy the average survival of patient’s only increased to 14.6 months (4, 5).

**Figure 1:**
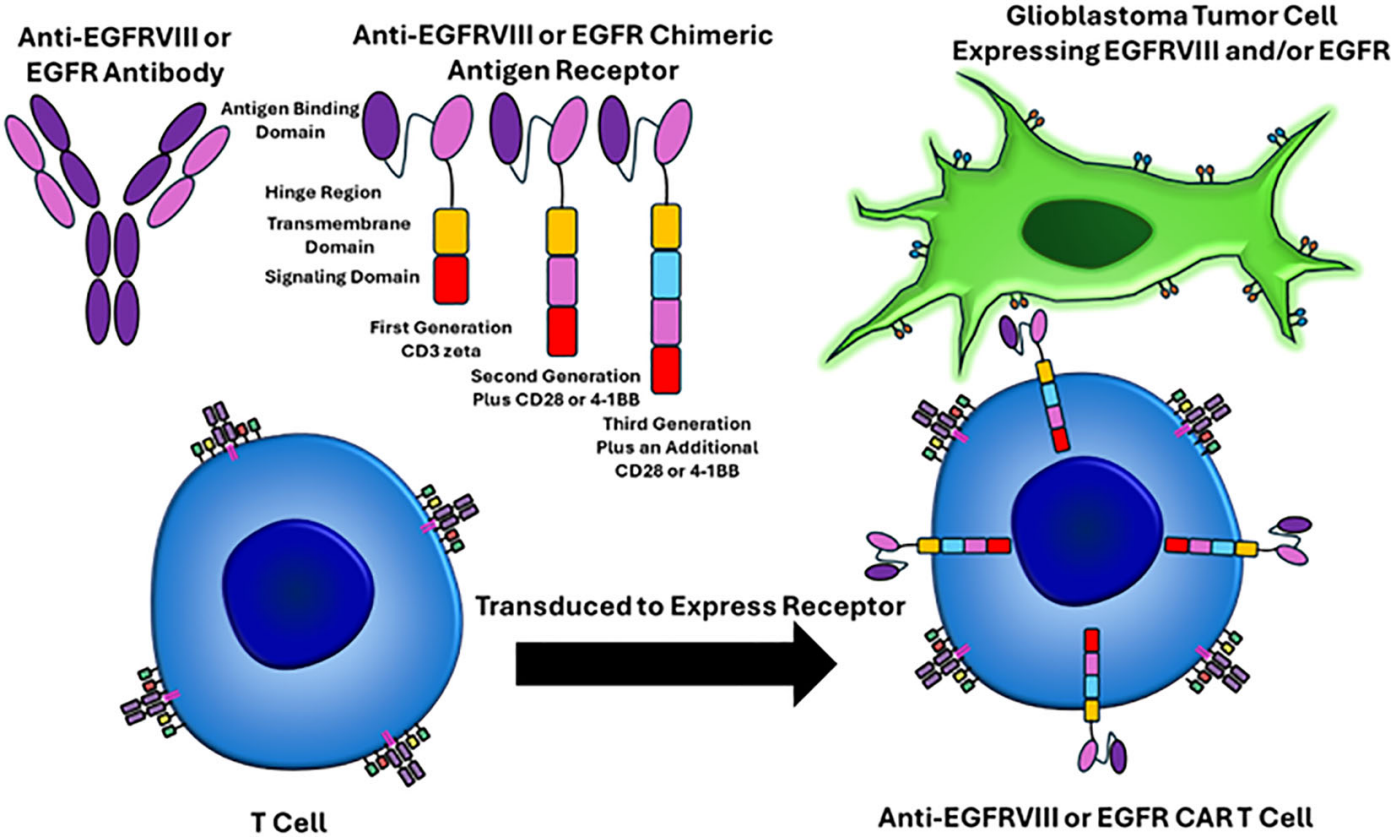
EGFRVIII and EGFR targeted CAR T cell mechanisms of action. The CAR T receptor targets EGFRVIII or EGFR on glioblastoma cells via single-chain variable fragments (antigen binding domain) that recognize epitopes on these proteins. The hinge region is the extracellular region that extends from the antigen binding domain to the transmembrane domain. The transmembrane domain functions as an anchor to the T cell membrane. One or more intracellular signaling domains are present. First generation CAR T cells only had a CD3 zeta chain and were not very effective. Second generation CAR T cells added an additional signaling domain, including CD28 or 4-1BB. Third generation CAR T cells have three intracellular signaling domains. T cells are harvested from the patient through apheresis, virally transduced to express the CAR T cell receptor, the CAR T cells are infused back into the patient, and then the CAR T cell can recognize the epitope of interest on the surface of the tumor cells.

Classically, GBM pathogenesis involves a multitude of cellular adaptations and signaling cascades that cultivate a proliferative and immunosuppressive tumor microenvironment (6, 7). Therefore, altering the tumor immuno-microenvironment and targeting the immune system to attack and eliminate cancer cells is an attractive approach. Recently, chimeric antigen receptor T-cell therapy is a revolutionary form of cellular immunotherapy, which has produced remarkably effective results in hematological malignancies (8–14). In short, chimeric antigen receptors are lymphocytes engineered to recognize and eliminate cells expressing specific antigens. CAR’s are most commonly T cells which consist of four main components (a) an extracellular antigen binding domain, which recognizes the target antigen of interest, (b) a hinge region, which is the extracellular region that extends the antigen binding domain from the transmembrane domain, (c) the transmembrane domain, which functions as an anchor to the T cell membrane, and (d) one or more intracellular signaling domains (8). **Figure 1** depicts the structure and mechanism of anti-EGFRVIII and EGFR CAR T cells.

In hematologic malignancies, for example, anti-CD19 CAR T cell therapy directed against B-cell malignancies was approved by the United States Food and Drug Administration in 2017 (11–13). Theoretically, this novel cancer treatment could be applied to solid tumors to potentially improve patient outcomes. Unfortunately, however, there are still several difficulties that must be addressed including limited efficacy against solid tumors due to multiple mechanisms, including but not limited to antigen escape, limited persistence, poor trafficking and tumor infiltration, and the immunosuppressive microenvironment (8, 9). In regard to GBM, multiple CAR T cell target antigens have been identified and CARs have been generated and tested in both preclinical and clinical studies (15, 16). One potentially exciting antigen is the epidermal growth factor receptor (EGFR) antigen, as it is frequently amplified in glioblastoma and a mutant variant is only expressed on the tumor (15–17). In this review, we will examine the preclinical and human study results of EGFR-targeted CAR T cell therapies and discuss current limitations and propose future directions.

## 2 EGFR epitope

EGFR is classically amplified and plays a key role in the progression and development of solid tumors including glioblastoma, non-small cell lung cancer, and breast, gastroesophageal, and colorectal cancer (18–20). In over 50% of GBMs, EGFR is amplified (17). Because of the high expression rate and role that EGFR plays in alteration of tumor development, progression, angiogenesis, invasion, and survival, EGFR is an exciting potential target for immunotherapy (4, 17).

## 3 EGFRVIII epitope

A common GBM associated variant of EGFR, Epidermal Growth Factor Receptor Variant III (EGFRVIII), is frequently expressed and correlates with the pathogenesis of GBM (15–17, 21, 22). More specifically, EGFRVIII is associated with tumor progression, invasiveness, and shorter survival (15–17, 21, 22). EGFRVIII is a result of an 801 base pair in-frame deletion from exons 2 to 7 (23–25).

## 4 EGFRVIII CAR T cells minimize on-target off-tumor toxicities

Importantly, the variant structure of the extracellular domain EGFR VIII has been shown to be able to be targeted by monoclonal antibodies in a specific manner, which reduces the risk of systemic on-target, off-tumor toxicities (26–28). Because of the fact that EGFRVIII is only expressed on the GBM, it is a promising target as theoretically CAR T cells targeting EGFRVIII would avoid one of the major limitations of CAR T cell therapy, on-target-off-tumor effects (26–28). The benefit of targeting the CARs to EGFRVIII is that this antigen is only expressed in the tumor. This significantly lowers the risk of toxicity when using multiple CARs. Consideration of toxicities because of off-target effects is critical and must be assessed clinically to minimize the potential devastating effects of toxicities. Because of this, multiple strategies have been used to develop CAR T cell therapies and vaccines targeting EGFRVIII (26–29). In initial preclinical studies, EGFRVIII demonstrated significant killing of tumor cells (29). For example, Miao et al. demonstrated that the survival rate of mice was significantly enhanced, the EGFRVIII CAR T cells proliferated, and the growth of tumor cells was reduced in mice treated with EGFRVIII targeted CAR T cells (30). All of these preclinical studies were exciting as they demonstrated excellent killing ability (29, 30).

Studies in human subjects and randomized control trials, unfortunately, have produced more heterogeneous results. The first clinical study involving intravenous administration of autologous CAR T cells targeting EGFR VIII for recurrent glioblastoma occurred in 2017 (28). O’Rourke et al. reported the findings of the first 10 cases of recurrent glioblastoma treated with EGFR VIII-targeted CAR T cells. In this study, 7 patients underwent surgical intervention. Post-surgical analysis of the tissue showed that EGFRVIII targeted CARs trafficked to the tissue, and importantly, there was significant antigen reduction observed in 5 of the 7 patients (28). NCT02209376, showed anti-tumor effective CAR T cells with a median survival of 8 months in all patients (28). A novel single-chain fragment variable (scFv) that targets EGFRVIII, GCTO2, was constructed by Abbott et al. and injected to assess the efficacy of GCTO2 EGFRVIII-targeted CAR T cells using the U87 EGFRV3 glioblastoma model (31). After a single intravenous injection of GCTO2 CAR T cells, significant regression of tumor cells was demonstrated suggesting a very high specificity for EGFRV3 (31). Further evaluation, however, showed that the affinity of this CAR was significantly lower compared to clone 2173 scFv (31). Notably, many other CARs targeting other antigens on GBM including CAR T cells for GBM therapy are ephrin type-A receptor 2 (EphA2) (NCT02575261), and mucin 1 (MUC1) (NCT02839954, NCT02617134) are currently being developed. In addition, IL13Rα2-targeted CAR T cells are being worked on for glioblastoma. A patient with multifocal glioblastoma that received IL13Rα2 CAR T cells showed regression of all observed tumors for 7.5 months with no grade 3 or higher toxic effects (32). A phase 1 clinical trial in patients with recurrent high-grade glioma receiving IL13Rα2-targeted CAR T cells showed stable disease or better being seen in 50% of the treated patients and was safe (33). However, extensive review of these targets is beyond the scope of this review.

## 5 Minimal immunologic toxicities associated with EGFRVIII CAR T cells

The most common adverse events seen in patients undergoing clinical trials with third generation CAR T cells targeting EGFR VIII were related to the lymphodepleting chemotherapy, which in patients treated with CAR T cell therapy is used before CAR T cell therapy infusion (28, 34, 35). Notably, 2 patients did have the side effect of severe dyspnea when CAR T cell therapy was administered at the highest dose with one of these patients ultimately dying (4, 28, 34). Several other patients had new minor changes in their neurological exam or seizures, which required treatment with antileptic medications or steroids (34). Importantly, however, no patients ended up developing significant side effects from an immunological standpoint, which historically has been a severely limiting side effect in hematologic malignancies (28). Although toxicities are less of a concern for EGFRVIII target therapies because of the high specificity of the antigen for the tumor cells itself, toxicities for CAR T cell therapy are an important limitation of CAR T cells in general and are a critical factor in the design of the CAR. Design of the antigen binding domain, hinge region, transmembrane domain, and signaling domains all can be optimized to potentially reduce toxicities and careful selection of dual antigens will be necessary to not only improve therapy but also continue to keep toxicity low.

## 6 EGFRVIII CAR T cells and the tumor microenvironment

Although there is exciting anecdotal data, including a patient with a history of recurrent GBM who received EGFRVIII targeted CAR T-cell therapy who ended up surviving for 36 months after treatment with CAR T cell therapy, these responses have not been repeatably observed in other patients (35). For instance, a pilot trial demonstrated that anti-EGFRVIII CAR T cells showed no significant clinical efficacy in treating GBM in a phase I pilot trial (34). Unfortunately, although EGFRVIII targeted CARs can be a useful target as they are relatively highly expressed and limit on-target off-tumor toxicities, the progression-free survival in some studies has only been a month while the overall median survival after treatment is only 6.9 months (34). Therefore, it is critical to understand that in order to get the most robust efficacy from EGFR targeted CAR T cells, the tumor microenvironment must also be targeted and manipulated. The immunosuppressive microenvironment is a key consideration as many cell types that drive immunosuppression including myeloid derived suppressor cells, tumor associated macrophages, and regulatory T cells can infiltrate solid tumors and drastically reduce the effectiveness of CAR T cell therapy as these cells produce tumor facilitating cytokines, chemokines, and growth factors. Strategies to ameliorate this immunosuppressive microenvironment include the utilization of immune checkpoint inhibitors with CAR T cell therapy. This investigation of immune checkpoint inhibitors with CAR T cell therapy is ongoing multiple types of solid tumors including gliomas. Combination immune checkpoint inhibitor and CAR T cell therapy has produced excellent results hematological malignancies with great potential in solid tumors. Although theoretically combining CAR T cell therapy targeting EGFRVIII with immune checkpoint therapy should significantly improve efficacy, a recent Phase I trial unfortunately demonstrated no efficacy in these patients, suggesting the need for additional strategies (36). In a preclinical study, metformin and rapamycin pre-treatment of EGFRvIII CAR-T cells improved metabolic states of the CAR T cells in the hypoxic microenvironment of glioblastoma and prolonged mouse survival (37). Other combination therapy strategies that have been attempted with CAR-T cells mainly in hematological malignancies and in some solid tumors include immunomodulators, BiTEs, vaccines, radiation, oncolytic virus, dendritic cell vaccines, cytokine or cytokine receptor blocker therapy, BTK inhibitor, PI3-kinase inhibitor, LAG3 inhibitor, dimerizer drug, stem cell transplantation, chemotherapy, and corticosteroids (38). Specifically, with CAR T cell combinations in solid tumors, strategies have included trying to increase tumor infiltration and tumor recognition with DDR inhibitors, angiogenesis inhibitors, MAPK inhibitors, and EZH2 inhibitors; trying to enhance cytotoxicity and reduce exhaustion with lenalidomide, TLR agonists, metabolic regulators, BET inhibitors, and DNMT inhibitors; and trying to increase memory phenotype with TET2 inhibitors, HDAC inhibitors, metabolic regulators, PI3K-AKT inhibitors, LCK/FYN inhibitors, and JAK inhibitors (39). Further study is necessary to tune the microenvironment to be more responsive to CAR T cell therapy. Efforts have also focused on engineering CARs that are resistant to immunosuppressive factors in the hostile tumor microenvironment such as TGF-beta (40). Many studies are also looking at how to create CARs with numerous cytokines to create armored CARs, which express pro-inflammatory cytokines instead of just focusing on inhibitory signals. Thus, further study is necessary to tune the microenvironment to be more responsive to CAR T cell therapy.

The tumor microenvironment presents a significant challenge to the field as it not only serves as a physical barrier but also is immunosuppressive. Thus, the tumor microenvironment prevents CAR T cells from properly trafficking and infiltrating solid tumors drastically reducing their efficacy. In order to obtain the efficacy seen in hematological malignancies, it will likely require multiple technical advances to circumvent the immunosuppressive microenvironment. Multiple approaches are currently being investigated to improve trafficking and infiltration. One such method involves the utilization of delivery routes other than systemic delivery. Currently, intraventricular injection and other local administration routes versus systemic delivery are currently under investigation (41). It is important to note that local delivery, including intraventricular administration, does not require lymphodepletion, thus avoiding the side effects discussed above. Additionally, local delivery allows for repeated administration, which is advantageous. Another exciting approach is to design CARs that express chemokine receptors that match and respond to tumor derived chemokines. These chemokine receptor expressing CARs have been shown to be possibly effective in CAR T cell therapies targeting other solid tumors and further investigation in glioblastoma is needed (42). Another strategy takes advantage of the fact that the tumor stroma extracellular matrix is composed of heparin sulfate proteoglycan (43). CAR T cell therapies can be engineered to express heparinases which function to degrade this physical barrier. These heparinase expressing CARs are currently under investigation and application to GBM is worth investigation. One possible approach to limit the immunosuppressive tumor microenvironment is to inhibit vascular endothelial growth factor, as inhibition of VEGF not only normalizes tumor vessels in both human and mouse models, but also improves the delivery of CD8 positive T cells and the effectiveness of immunotherapies. Excitingly, a recent study found that treatment with anti-mouse VEGF antibodies improved both the distribution and infiltration of CAR T cells targeting EGFR VIII in GBM, which resulted in both prolonged survival and slowed tumor growth compared to EGFR VIII CAR T cell therapy by itself (44).

## 7 EGFRVIII CAR T cells and antigen escape

As with many solid tumors, targeting one antigen using CAR T cell therapy is currently insufficient to produce the drastic responses seen in hematological malignancies. The current limitations of CAR T cell therapy’s efficacy against solid tumors including glioblastoma are due to multiple mechanisms of tumor resistance to single-targeted CAR T cell construct. One important consideration is the mechanism of antigen escape. Although initially tumor cells may express high levels of an antigen, CAR T cell therapies targeting these cells will result in either partial or complete loss of the target antigen expression as the CAR eliminates these cells from the tumor. In other words, the CAR is naturally selecting tumor cells without the antigen by killing those with the antigen. A strategy to circumvent this limitation is to use dual-targeted CAR T cell therapy in which chimeric antigen receptors targeting two or more antigens are use. These antigens can be targeted by using two CAR T cells that each target a single antigen or through the use of tandem CARs, which is a single CAR construct that contains two single-chain variable fragment antigen recognition domains in order to concomitantly target multiple target tumor antigens (45–47). Tandem CAR T-cells targeting EGFRVIII and IL-13R α2 appear to be a potentially attractive approach based off preclinical data using patient-derived xenograft mouse models, which demonstrated long term durable and complete responses against heterogeneous GBM tumors (48). A syn Notch receptor that recognizes a specific priming antigen, such as the heterogeneous but tumor-specific glioblastoma neoantigen epidermal growth factor receptor splice variant III (EGFRvIII) or the central nervous system (CNS) tissue-specific antigen myelin oligodendrocyte glycoprotein (MOG), locally induces expression of a CAR (49). This allows for killing by targeting antigens that are homogeneous but not absolutely tumor specific and reduces exhaustion (49). EGFRvIII synNotch-CAR T cells showed higher antitumor efficacy and T cell durability than their regular CAR T cell counterparts without off-tumor effects (49). T cells with a synNotch-CAR circuit primed by MOG also resulted in good control without priming outside the brain (49).

## 8 EGFR CAR T cells for glioblastoma

An unintended consequence of EGFRVIII-targeted CARs, however, is that it can lead to the proliferation of EGFRVIII-negative-EGFR-positive glioblastoma, an example of antigen escape in action (50). An exciting potential solution to this limitation is the creation of CAR T bispecific T cell engager (BiTE) cells (50). These CAR-T BiTE cells have the ability to secrete EGFR-specific BiTEs, which have shown the ability to eliminate heterogeneous tumors in glioblastoma mouse models through recruiting untransduced T cells against wild-type EGFR and redirecting CAR T cells (50). Excitingly, this approach also appears to not produce the toxicity seen with EGFR-targeted CAR T cells (50). In a first in human study, three recurrent glioblastoma patients were treated with CARv3-TEAM-E T cells, which are chimeric antigen receptor (CAR) T cells engineered to target the epidermal growth factor receptor (EGFR) variant III tumor-specific antigen, as well as the wild-type EGFR protein, through secretion of a T-cell-engaging antibody molecule (TEAM) (51). The treatment appeared to be safe. There was regression of the tumor, but this was transient in 2 of 3 patients (51). Another preclinical model involving human-induced pluripotent stem cell-derived teratoma xenograft model showed that EGFR806 CAR T cells, which are able to recognize low density EGFR, both selectively and effectively targeted EGFR-expressing tumor cells (52). Similarly, Chen et al. showed that an EGFR806 targeting CAR was effective *in vitro* and *in vivo* in a dose dependent manner (29).

Excitingly, interim results of a Phase I clinical trial assessing intrathecal efficacy of bivalent CAR-T cells targeting EGFR and IL-13R-alpha-2 in six recurrent glioblastoma patients demonstrated improvements in tumor size and enhancement, substantial CAR T cell abundance, and significant cytokine release in the cerebrospinal fluid in all six patients (53). These results are promising; however, longer follow-up time and a higher sample size are needed for more definitive conclusions. HER2 also appears to be an effective co-target in glioblastoma (54, 55). Other studies have used CARs targeting HER2 and IL-13RA2 in glioblastoma and produced some initial promising results (56). Further studies using EGFR-directed CAR T cells in combination with other targeted antigens are warranted in the future.

A potential limitation of using EGFR-targeted CAR T cells is on-target, off-tumor effects as EGFR is more widely expressed throughout the body than the more specific EGFVIII and thus a higher chance of CAR T associated toxicities ensues. The tumor microenvironment and antigen escape remain challenges with EGFR as a therapeutic target as well.

## 9 Discussion

EGFRVIII and EGFR as CAR T cell targets are exciting as they are commonly amplified in glioblastoma. A summary of the studies covered in this review are shown in **Table 1**. Because of the fact that EGFRVIII is only expressed on the GBM, it is a promising target as theoretically CAR T cells targeting EGFRVIII would avoid one of the major limitations of CAR T cell therapy, on-target-off-tumor effects (26–28). A potential limitation of using EGFR-targeted CAR T cells is on-target, off-tumor effects as EGFR is more widely expressed throughout the body than the more specific EGFVIII and thus a higher chance of CAR T associated toxicities ensues. The EGFRVIII has theoretical potential to be a fantastic and an efficacious target as it is only expressed on tumor cells. Although pre-clinical studies demonstrate remarkable killing, human studies unfortunately have produced heterogeneous results that suggest further optimization and strategies are needed in order to continue to push CAR T cell therapy towards improving patient outcomes. The tumor microenvironment presents a significant challenge to the field as it not only serves as a physical barrier but also is immunosuppressive. Thus, the tumor microenvironment prevents CAR T cells from properly trafficking and infiltrating solid tumors drastically reducing their efficacy. The immunosuppressive microenvironment is a key consideration as many cell types that drive immunosuppression including myeloid derived suppressor cells, tumor associated macrophages, and regulatory T cells can infiltrate solid tumors and drastically reduce the effectiveness of CAR T cell therapy as these cells produce tumor facilitating cytokines, chemokines, and growth factors. Although theoretically combining CAR T cell therapy targeting EGFRVIII with immune checkpoint therapy should significantly improve efficacy, a recent Phase I trial unfortunately demonstrated no efficacy in these patients, suggesting the need for additional strategies (36). As with many solid tumors, targeting one antigen using CAR T cell therapy, including targeting EGFRVIII or EGFR, is currently insufficient to produce the drastic responses seen in hematological malignancies due to antigen escape. Thus, while targeting EGFRVIII or EGFR in glioblastoma via CAR T cell therapy holds promise, there are still many obstacles to be overcome before these strategies can become effect clinical treatments.

**Table 1:** Summary of EGFRVIII and EGFR CAR T cell studies in glioblastoma.

Publication	Study Type	Target	CAR T Cell Construct	Outcomes	Challenges/Solutions
Miao et al. (30)	Preclinical	EGFRVIII	GFRvIII^+^ CAR (humanized 139 anti-human EGFRvIII single-chain variable fragment in tandem with the hCD28-41BB-CD3 zeta chain signaling domain)	Effective *in vitro* and prolonged survival *in vivo*, but mice eventually succumb to disease.	Diminished long term efficacy.
Abbott et al. (31)	Preclinical	EGFRVIII	GCT02 scFv, human CD8α hinge, human CD28 transmembrane and costimulatory domains, human CD3ζ T cell signaling domains	Effective *in vitro* and *in vivo*.	Had a modest cytokine profile but good tumor killing, may be good to prevent toxicities.
Dong et al. (44)	Preclinical	EGFRVIII	clone 139 scFv for EGFRvIII CAR), mouse CD8a hinge and transmembrane domain, CD28 costimulatory domain, CD3ζ intracellular domain	CAR T cell efficacy was enhanced by anti-VEGF therapy.	Improves infiltration of the CAR T Cells and alters the tumor microenvironment.
Schmidts et al. (48)	Preclinical	EGFRVIII, IL-13Rα2	hGM-CSF or CD8 leader sequence, a CD8 transmembrane (TM) domain, a 4-1BB costimulatory domain, and a CD3-zeta signaling domain, scFv target IL-13Rα2 membrane tethered IL-13 ligand mutated at E13Y, which allows the zetakine to bind more specifically to IL-13Rα2 and scFv CARTEGFRvIII contains a humanized scFv that is specific for EGFRvIII	Tandem CAR T cells showed enhanced *in vitro* and *in vivo* efficacy compared to single antigen targeting CAR T cells.	Tandem CAR T cells help reduce antigen escape.
Hatae et al. (37)	Preclinical		scFv derived from human EGFRvIII-specific monoclonal antibody 3C10, CD8α transmembrane domain, CD28 and 4-1BB costimulatory domains, and CD3ζ domains.	Metabolic states of CAR-T cells in hypoxic conditions were improved by pre-treatment of CAR T cells with metformin and rapamycin, and this extended the survival of mice.	Metformin and rapamycin pre-treatment of EGFRvIII CAR-T cells improved metabolic states of the CAR T cells in the hypoxic microenvironment and enhanced mouse survival.
O’Rourke et al. (28)	Clinical, phase 1	EGFRVIII	scFv derived from human EGFRvIII-specific monoclonal antibody 3C10, CD28 trans-membrane, and intracellular domains as well as the 4-1BB ICD and CD3ζ domains.	Manufacturing and infusion of EGFRvIII CAR T cells are feasible and safe, without evidence of off-tumor toxicity or cytokine release syndrome.	Although intravenous infusion results in on-target activity in the brain, overcoming the adaptive changes in the local tumor microenvironment and addressing the antigen heterogeneity may improve the efficacy of EGFRvIII-directed strategies in GBM.
Goff et al. (34)	Clinical, phase 1	EGFRVIII	scFv human monoclonal Ab 139, CD8 linker domain, CD28 and 4–1BB costimulatory domains, and CD3ζ signaling domain	Treatment with EGFRVIII CAR T cells did not demonstrate clinically meaningful impact in patients with glioblastoma multiforme.	The paucity of safe normal self-proteins or tumor-specific mutated antigens to target on the surface of tumor cells is a severe limitation in the more widespread application of CAR T technology for solid cancers including in glioblastoma.
Durgin et al. (35)	Clinical, case report	EGFRVIII	Humanized anti-EGFRvIII single-chain variable fragment fused to the hinge and transmembrane domain of CD8 and the human 4-1BB and CD3ζ intracellular signaling domains	A patient received a single peripheral infusion of CAR T-EGFRvIII cells and survived 36 months after disease recurrence, exceeding expected survival for recurrent glioblastoma.	CAR T-EGFRvIII cells persisted in the patient’s peripheral circulation during 29 months of follow-up, the longest period of CAR T persistence reported in GBM trials to date, unfortunately this was only seen in one patient.
Bagley et al. (36), (*Nat Cancer*)	Clinical, phase 1	EGFRVIII	Humanized anti-EGFRVIII scFv fused to hinge and transmembrane domain of CD8 and the human 4-1BB and CD3ζ intra cellular signaling domains	Treatment with EGFRVIII CAR T cells and PD1 inhibition in glioblastoma is safe and biologically active but lacks efficacy.	The tumor microenvironment remains an obstacle to overcome in glioblastoma and anti-PD1 therapy appears to not be sufficient to prevail.
Chen et al. (29)	Preclinical	EGFRVIII and activated EGFR	806 scFv, human CD28 costimulatory domains, human CD3ζ T cell signaling domains	Effective *in vitro* and *in vivo* in a dose dependent manner.	Dose dependent efficacy.
Choi et al. (50)	Preclinical	CAR targeting EGFRVIII that also secretes BiTE specific to EGFR	CART.BiTE constructs contain a CD8 transmembrane domain in tandem with an intracellular 4-1BB costimulatory domain and CD3ζ signaling domain with an anti-EGFRVIII scFv for the CAR and anti-EGFR and anti-CD3 scFv for the BiTE	CART.BiTE cells showed improved efficacy against EGFRVIII/EGFR heterogenous tumors compared to anti-EGFRVIII CAR T cells in mouse models.	While EGFR-specific CAR T cells showed toxicity, CART.BiTE cells did not *in vivo*.
Choi et al. (51)	Clinical, phase 1	CAR targeting EGFRVIII tumor-specific antigen and a T-cell-engaging antibody molecule (TEAM), targeting wild-type EGFR	CARv3-TEAM-E lentiviral vector with EGFRVIII targeting scFv, a 4-1BB costimulatory domain linked to CD3ζ, an EGFR TEAM following a ribosomal skip element, and a truncated CD19 as a surface marker for transduction.	The treatment appeared to be safe. There was regression of the tumor, but this was transient in 2 of 3 patients.	Need to enhance duration of treatment effect.
Ravanpay et al. (52)	Preclinical	EGFRVIII and activated EGFR	806 scFv, human 4-1BB costimulatory domains, human CD3ζ T cell signaling domains	EGFR806-CAR T cells effectively and selectively target EGFR-expressing tumor cells *in vitro* and *in vivo*.	The role of the tumor microenvironment and survival of EGFR negative malignant cells remain areas of concern for this strategy.
Bagley et al. (53), (*Nat Med*)	Clinical, phase 1	EGFRVIII and activated EGFR, IL13Rα2	Humanized EGFR epitope 806, a cryptic, conformational epitope predominantly accessible with dysregulation of EGFR activation due to over expression and/or the presence of EGFR extra cellular domain mutations and IL13Rα2 targeting scFv, transmembrane domain, human 4-1BB and CD3ζ intracellular signaling domains	Preliminary safety and bioactivity and encouraging early efficacy signal was detected and requires additional study.	Patients experienced some neurotoxicity. Patients had cytokine release syndrome in CSF.
Choe et al. (49)	Preclinical	Syn Notch receptor recognizing EGFRvIII or MOG with an inducible CAR recognizing the elements described in the next column.	CARs were built by fusing EphA2 scFv, IL13 mutein [E13K,K105R], or IL13 mutein [E13K,K105R]-G4Sx4-EphA2 scFv to the hinge region of the human CD8α chain and transmembrane and cytoplasmic regions of the human 4–1BB, and CD3z signaling domains. SynNotch receptors were built by fusing α-EGFRvIII 139 scFv, α-MOG M26 scFv, or α-CDH10 scFv (Sidhu laboratory) to mouse Notch1 (NM_008714) minimal regulatory region (res.1427–1752) and Gal4 DBD VP64.	The syn Notch receptor that recognizes a specific priming antigen (EGFRvIII or MOG) and induces local expression of the CAR resulting in for tumor focused killing, high antitumor efficacy, and reduces exhaustion.	Results in improved tumor focal killing and less exhaustion.
